# The Recovery Deficit: A Simulation Model of Physician Performance Under Sleep Deprivation

**DOI:** 10.7759/cureus.95729

**Published:** 2025-10-30

**Authors:** Joshua Moen

**Affiliations:** 1 School of Community Medicine, University of Oklahoma Health Sciences Center, Tulsa, USA

**Keywords:** clinical errors, medical residency, performance optimization, quality of sleep, resilience and well-being

## Abstract

Background: Medical education policy has focused on duty hours as the primary lever for safety and training quality. This work-centric framing treats sleep as residual time rather than a prerequisite for learning and safe practice. As a result, trainees often operate in chronic sleep debt, accumulating a performance and safety deficit that duty-hour counts alone cannot resolve.

Objective: This study uses Monte Carlo simulation to quantify how sleep deprivation affects clinician performance, safety, and well-being and establishes evidence-based sleep visualizations to promote recovery-centered residency redesign.

Design, setting, and participants: Computer-based Monte Carlo simulation (Stata 18.0), parameterized from experimental and cohort literature on sleep restriction and clinical performance. Each of 500 iterations generated 11,000 synthetic clinician-days uniformly across medical students, residents, and attending physicians. Nightly sleep was the primary exposure; outcomes included reaction time, clinical and diagnostic accuracy, attentional lapses, medical errors, mood, and burnout. Outputs were baselined to the ≥8 h sleep category to visualize the deficit relative to a rested state.

Results: Performance declined steeply with shorter sleep. Composite score fell from 83.3 (≥8 h) to 56.8 (4 h), 38.9 (2 h), and 9.0 (0 h). Reaction time slowed from 280 ms (≥8 h) to 404 ms (4 h) and 602 ms (0 h). Error risk rose markedly: <4 h vs ≥4 h, 16.2% vs 6.8% (OR: 2.62; risk difference +9.3 pp); <6 h vs ≥6 h, 9.7% vs 6.0% (RR: 1.62; OR 1.68; risk difference +3.7 pp). Burnout prevalence (modeled probability) increased from 12.8% (≥8 h) to 51.3% (0 h). Uncertainty was summarized using simulation percentiles across iterations.

Conclusions and relevance: Duty-hour counts alone obscure a daily performance and safety deficit driven by inadequate recovery sleep. Quantifying and visualizing this deficit shows severe, dose-dependent impairments and elevated error risk with short sleep, supporting a recovery-first training model that designs duty hours around protected seven to nine hours of sleep to enhance patient safety, learning, and clinician well-being.

## Introduction

American medical training has long been defined by a central tension: the belief that immersive experience creates competent physicians versus the risk of fatigue such immersion creates. For over a century, the Halstedian model equated endurance with dedication, embedding the narrative that long hours are necessary for excellence [1]. Subsequent regulatory reforms, largely focused on duty hours [2,3], have produced mixed effects, suggesting that time‑on‑the‑clock is an incomplete solution [4,5].

A persistent assumption is that enduring sleep deprivation cultivates toughness. This paper argues the opposite: chronic deprivation undermines resilience by sabotaging the neurological processes required for learning and skill acquisition [6,7]. Residents average only six hours of sleep per day [8,9], leaving them in chronic sleep debt. The 2009 Institute of Medicine (IOM) report represented a necessary intervention, recommending sweeping changes to improve resident health, safety, and performance [10]. However, the reforms that followed failed to move beyond the traditional framework of manipulating duty hours. The focus on time restrictions alone, after producing inconclusive results [11], helps explain why the Halstedian model persists.

The landmark iCOMPARE trial epitomizes this approach. The trial's non-inferiority design compared two sleep-deprived groups, "flexible shifts" versus "standard shifts", thereby accepting sleep restriction as inevitable rather than testing whether consistent, adequate sleep improves outcomes. Moreover, both trial arms averaged <7 hours of sleep on day shifts, with shorter sleep durations showing decreased alertness and increased performance lapses in a dose-dependent fashion [12]. Comparing two sleep-restricted groups cannot study paths to optimize resident health, safety, and performance as the IOM recommended.

Elite athletics [13], aviation [14], and military operations [15] each demonstrate the shift from a "sleep is for the weak" mentality to evidence-based recognition that sleep is essential for optimal performance. Within medicine, anecdotal arguments persist that promote long shifts and endurance as a rite of passage, fostering toughness and resilience. Challenges to this mentality are often dismissed as advocacy for "weakness" or reduced rigor. Paradoxically, by promoting chronic sleep deprivation, extended shifts, and other endurance-based rites of passage, medical training severely limits the efficiency of the hard work it demands [16,17].

A recovery‑centric model is therefore needed, one that aligns training with performance science. Built upon the neurobiological foundation of sleep, such a system could transform residency from a rite of endurance to an architecture of optimization. To provide a quantitative foundation for this proposed architecture, we developed a Monte Carlo simulation to model the dose-response effects of nightly sleep on clinician performance, safety, and well-being, establishing evidence-based visualizations to guide recovery-centered training reform.

## Materials and methods

Study design and overview

We developed and executed a computer-based Monte Carlo simulation in Stata 18.0 (College Station, TX) to estimate dose-response relationships between nightly sleep duration and clinician performance, safety, and well-being. All data were synthetic and parameterized from published experimental and cohort literature on sleep restriction, attentional lapses, reaction time, clinical and diagnostic performance, errors, mood, and burnout. Laboratory dose-response anchors for reaction time and attentional lapses were taken from controlled sleep-restriction and deprivation paradigms [18-20], with broader cognitive effects informed by meta-analytic syntheses [21], and clinician performance studies [22,23]. Error-risk calibration drew on large cohort evidence relating short sleep and extended-duration shifts to increased medical errors [9,24,25], and well-being parameters were informed by associations between sleep loss, mood, and burnout [3,5,8,26]. To provide clinically interpretable metrics, key outputs, including a composite performance score, were expressed as a percentage of a rested baseline defined as the mean performance among observations with ≥8 hours of sleep. A companion, public-facing visualization, the Medical Performance Sleep Calculator, implements the same mappings and reports reaction-time impairment with blood alcohol concentration equivalence derived from Arnedt et al. [27] and Williamson et al. [28].

Population, exposure generation, and simulation scope

Each of R = 500 Monte Carlo iterations generated N = 11,000 synthetic daily observations representing individual clinicians, with professional roles uniformly assigned as medical student, resident, or attending physician. Nightly sleep hours were drawn from a truncated normal distribution (mean: 5.8 hours, standard deviation: 1.2 hours, bounded 0-9 hours), reflecting observed sleep distributions among trainees and healthcare workers [8,29,30]. Sleep debt, d, was defined as max (0, 8 − sleep hours), adopting an eight-hour restorative reference. To ensure adequate representation of extreme acute deprivation often observed during extended shifts and overnight call, 1,000 observations per iteration were explicitly set to 0 hours of sleep (d = 8), aligning the synthetic tail with prospective evidence on extended-duration work and safety outcomes [25,31]. Results were stable by approximately R = 300-500; we used R = 500 to balance precision and runtime.

Parameterization and stochastic structure

To propagate literature uncertainty, we sampled a fresh set of dose-response coefficients for each Monte Carlo iteration from normal priors centered on published estimates with standard deviations reflecting reported ranges. The mean slope for reaction time was +32 milliseconds per hour of sleep debt (approximate range: 27-37), anchored to controlled restriction paradigms and clinician reaction-time studies [18,20,21,23]. Clinical skills and diagnostic accuracy had mean slopes of −3.0 and −2.5 percentage points per hour, respectively, with quadratic moderation to capture nonlinearity at higher debts, reflecting decrements reported in applied clinical performance under fatigue [7,21-23]. Medical error propensity was modeled on the log-odds scale with a mean coefficient of +0.11 per hour of sleep debt (range: ~0.095-0.14), calibrated to cohort evidence linking short sleep and extended shifts to higher error risk [9,24,25]. Burnout propensity increased by +0.25 log-odds per hour (range: 0.20-0.30), and mood declined by approximately two points per hour (range: −2.5 to −1.5), consistent with observed sleep-well-being associations [5,6,24,26]. Attentional lapses followed a Poisson process whose log-rate included a quadratic term with mean 0.05 (range: 0.03-0.07), reproducing the exponential rise in lapses under cumulative restriction and deprivation [18-20]. To capture the shared physiological basis of fatigue across domains, we introduced correlated disturbances via multivariate normal draws with off-diagonal correlations between 0.3 and 0.5 across reaction time, diagnostic accuracy, clinical skills, lapses, error propensity, mood, and burnout, mirroring co-fluctuations observed in laboratory and field settings [18-21].

Outcome equations and modifiers

For each observation i with sleep debt di, we specified the following outcome equations: reaction time (ms), RTi = 280 + (bRT + role/specialty modifiers)·di + 1.0·di² + 20·zRT; clinical skills (%), Clin_i = 95 + (bClin + specialty/role modifiers)·di − 0.2·di² + 4·zClin; diagnostic accuracy (%), Diag_i = 90 + (bDiag + role modifiers)·di − 0.3·di² + 3·zDiag; attentional lapses drawn from Poisson(exp(−1 + 0.25·di + bLapQuad·di² + 0.4·zLap)); medical error probability given by logit−1(−2.94 + bErr·di + 0.01·di² + 0.3·zErr) and burnout probability by logit−1(−1.95 + bBurn·di + 0.3·zBurn); and mood (0-100), Mood_i = 80 + bMood·di − 0.2·di² + 5·zMood. Role and specialty modifiers operationalized experience-based compensation and task-specific vulnerabilities documented in the literature, with small compensatory effects for attending physicians and modest decrements for medical students [7,22,23].

Scaling, composites, aggregation, and baselining

To harmonize heterogeneous units and support interpretation, outcomes were normalized to 0-100 scales, with higher values indicating better functioning. Reaction time was inverted and linearly mapped so that 200 ms corresponded to 100 and 600 ms to 0. Clinical skills and diagnostic accuracy were linearly rescaled over plausible clinical ranges (60-100% and 70-100%, respectively). Attentional lapses were inverted and scaled to a fixed, precomputed 99th percentile cap estimated from a large reference draw to stabilize scores against heavy-tailed Poisson realizations, consistent with the exponential behavior of lapses under sleep loss [19,20]. Burnout was recorded as 100·(1 − burnout), and mood was bounded to the 0-100 range. A composite overall performance score was defined as the unweighted mean of four normalized domains, namely, clinical skills, diagnostic accuracy, reaction time, and attentional vigilance (inverse lapses), to reflect multidimensional cognitive and psychomotor impacts of sleep loss documented in experimental and clinical studies [21-23]. Within each iteration, outcomes were collapsed by integer sleep-hour categories (0 h, 1 h, …, ≥8 h), and results were then pooled across iterations to compute means and the 3rd and 97th percentiles for each sleep category. The rested baseline was defined as the mean composite for the ≥8h category, and all category means and bounds were re-expressed as percentages of this baseline to yield performance deficits comparable to laboratory dose-response curves [20,21].

Calibration, validation, and linkage to the public tool

Automated calibration checks were executed after pooling to confirm face validity and concordance with published evidence. First, the composite score at ≥8 hours of sleep was verified to reflect a high-functioning rested state on the 0-100 scale. Second, the mean medical error rate near four hours of sleep was inspected for agreement with cohort estimates showing elevated error risk under short sleep and extended-duration shifts [9,24,25]. Third, total sleep deprivation (0 hours) yielded severe impairment with floor behavior in the composite and exponential increases in lapses, reproducing classic deprivation effects [19,20]. An optional large-N fixed-parameter run (n = 200,000) provided sanity checks for rested reaction time distributions, extreme-lapse behavior near total deprivation, and the recovered error log-odds per hour of sleep debt against the targeted prior mean [9,20,24]. The public Medical Performance Sleep Calculator implements the same dose-response mappings and displays blood alcohol concentration-equivalent reaction-time impairments based on Arnedt et al. [27] and Williamson et al. [28], alongside the predicted overall performance, reaction time, clinical skills, diagnostic accuracy, attentional vigilance, mood, and burnout probability, with an explicit performance warning when predicted impairment may affect patient safety.

## Results

Across 500 Monte Carlo iterations (11,000 clinician-days each), we observed a clear dose-response degradation in performance and well-being as nightly sleep declined (Table 1).

**Table 1:** Simulated Performance and Wellness Outcomes by Hours of Sleep Data represent mean values from 500 Monte Carlo iterations, each simulating 11,000 daily observations of medical professionals (medical students, residents, and attending physicians). Sleep hours were sampled from a truncated normal distribution (mean: 5.8 h, SD: 1.2 h, range: 0-9 h) with 1,000 observations per iteration fixed at 0 hours to capture extreme deprivation scenarios. Performance metrics are population averages across all professional roles. Composite performance score represents the unweighted mean of four normalized domains: clinical skills, diagnostic accuracy, reaction time, and attentional vigilance (inverse of lapses). All performance measures are scaled 0-100, with higher scores indicating better function.

Hours of Sleep	Composite Performance (0–100)	Reaction Time (ms)	Clinical Accuracy (%)	Diagnostic Accuracy (%)	Mean Lapses	Medical Error Rate (%)	Mood Score (0–100)	Burnout Prevalence (%)
0 h	9	602	57.6	50.8	90.8	19.9	51.3	51.3
1 h	29.8	524	67.5	62.2	16.4	14	59.3	41.4
2 h	38.9	482	72.7	67.9	6.9	11.7	63.6	36
3 h	47.7	442	77.5	73.1	3.3	9.8	67.3	30.6
4 h	56.8	404	81.9	77.7	1.8	8.3	70.7	25.9
5 h	65.2	368	85.9	81.7	1	7.2	73.7	21.6
6 h	72.6	334	89.6	85.3	0.7	6.3	76.3	17.9
7 h	79.3	301	92.9	88.3	0.5	5.6	78.6	14.8
≥8 h	83.3	280	95	90	0.4	5.2	80	12.8

The composite performance score averaged 83 at ≥8 hours and decreased progressively with shorter sleep, reaching 65 at five hours, 57 at four hours, 39 at two hours, 30 at one hour, and ~9 at total deprivation. Psychomotor speed slowed substantially with restriction, with reaction times rising from a rested 280 ms to 404 ms at four hours and 602 ms at 0 hours, while clinical and diagnostic accuracy deteriorated in parallel (clinical: 95% at ≥8 h to 82% at four hours and 58% at 0 hours; diagnostic: 90%-78% and 51%). Attentional lapses exhibited the characteristic exponential pattern, remaining low above ~6 hours but increasing sharply with severe restriction (mean: 1.8 at four hours, 16.4 at one hour, 90.8 at 0 hours). Error risk rose meaningfully at common short-sleep thresholds: compared with ≥6 hours, sleeping <6 hours yielded a 62% higher risk (9.7% vs 6.0%; RR: 1.62), and the highest risk concentrated below four hours (16.2% vs 6.8% for <4 h vs ≥4 h; OR: 2.62, RD: 9.3 percentage points). Wellness outcomes worsened in tandem with sleep loss, with mood declining from 80 at ≥8 hours to 67 at three hours, 63.6 at two hours, 59.3 at one hour, and 51.3 at 0 hours, and burnout prevalence rising from 12.8% at ≥8 hours to 21.6% at five hours, 30.6% at three hours, 36-41% at two to one hours, and 51.3% at 0 hours. By construction, ~9% of days represented extreme deprivation (0 hours), producing severe impairment across domains and reinforcing the steep, clinically relevant risks when recovery sleep is absent. The companion Medical Performance Sleep Calculator implements identical dose-response mappings with role-specific adjustments. The calculator allows users to input sleep duration and other characteristics to see real-time predicted performance outcomes. The tool is also available at https://ucdhealthjm.github.io/Performance-Calculator/.

## Discussion

The findings of this simulation challenge the conventional narrative that equates endurance with competence in medical training, instead framing performance degradation as a perpetual operating deficit. When medical training schedules preclude seven to nine hours of nightly sleep, a figure consistent with widespread observations of residents [8,21,22], trainees are forced into a damaging cycle. During duty hours, they are learning and practicing while cognitively hindered [7] and physiologically handcuffed, which compromises both the safety of their work and the quality of their learning, consistent with meta-analytic evidence on the broad cognitive impacts of sleep loss [13]. Critically, they then miss the only biological window (restorative sleep) for the neural consolidation required to convert the day's experiences into durable skill, a process fundamentally dependent on sleep [6]. The current system, therefore, mandates impaired practice, evidenced by increased medical error rates under sleep restriction [9,16,17], and then blocks the learning from that practice from becoming permanent.

This perpetual deficit state is a direct product of an intense, decades-long focus on a single metric: the number of duty hours. Landmark trials such as iCOMPARE and the FIRST trial, while important, exemplify this narrow framing by comparing one model of sleep restriction against a slightly less restrictive one [3]. Such approaches, along with duty-hour reforms that have produced mixed effects on wellness and patient outcomes [4,5], essentially ask which degree of deficit is more tolerable. This line of inquiry fails to question the fundamental premise that chronic sleep debt is a necessary, or even acceptable, component of medical education. It debates the severity of the problem rather than establishing the foundation for a solution.

We argue for a complete inversion of this paradigm. Sleep must not be treated as the time leftover after work; it must be the non-negotiable foundation upon which a safe and effective training model is built. The guiding question for residency programs must change from "How many hours can a trainee work?" to "Given a protected 7-9 hours for recovery, how do we design the most efficient and impactful training experience in the time that remains?". This recovery-centric model shifts the goal from endurance to the optimization of learning, well-being, and patient safety. This promotes an ergogenic training model based on robust evidence, moving beyond simply mitigating harm to actively enhancing performance, a stark contrast to the decrements documented under typical training conditions [14,15].

The online calculator developed alongside this simulation was designed specifically to make this operating deficit tangible. By visualizing the daily impairments in reaction time and accuracy and contextualizing them with metrics such as blood alcohol concentration (BAC) equivalence [19,20], the tool makes the consequences of the current model undeniable. It reframes fatigue as a systemic, structural issue, supported by cohort studies linking short sleep to burnout and medical errors [9,16], not an individual failing of resilience. True reform requires designing educational systems where adequate recovery is a structural prerequisite for the work of becoming a physician.

Limitations

This study has several limitations that should be acknowledged. First and foremost, as a Monte Carlo simulation, our study relies on synthetic data parameterized from existing literature. Its validity is therefore contingent on the accuracy and generalizability of the source studies used for calibration. Although we incorporated uncertainty by sampling parameters from distributions, the model simplifies complex, nonlinear physiological relationships.

Second, the model simplifies complex human physiology and does not account for individual heterogeneity in sleep needs or resilience to sleep deprivation. Factors such as chronotype, underlying health conditions, and genetic predispositions can moderate the impact of sleep loss but were not explicitly modeled.

Third, our simulation models a direct dose-response relationship between sleep and various outcomes but does not incorporate other critical confounders prevalent in the clinical environment, such as workload intensity, case complexity, interpersonal stress, or institutional safety culture. These factors undoubtedly interact with fatigue to influence performance and well-being.

Finally, our composite "Overall Performance Score" uses an unweighted average of four distinct domains (clinical skills, diagnostic accuracy, reaction time, and attentional vigilance). This simplification may not reflect the task-dependent importance of different cognitive domains in clinical practice, where, for instance, diagnostic accuracy may be more critical than reaction time in one scenario but not another. Despite these limitations, the simulation provides a quantitative framework for conceptualizing the dose-response effects of sleep loss and highlights the urgent need for a paradigm shift in medical education. While our model is parameterized with empirical data from residency populations and grounded in validated sleep science, the projected outcomes of recovery-centered policy reforms require prospective validation in real-world training environments.

## Conclusions

The long-standing focus on duty hours has fundamentally misdiagnosed the challenge of medical training by treating sleep as a cost rather than a prerequisite for learning. Our simulation demonstrates that safe practice and effective, durable learning are not products of endurance, but of an optimized cycle of work and recovery. This requires a paradigm shift from a work-centric to a recovery-centric model, where training is built upon a non-negotiable foundation of seven to nine hours of sleep. The future of medical education must therefore move beyond the simple arithmetic of the 80-hour workweek and embrace a physiologically informed approach that optimizes for safety, well-being, and mastery.
