# Correction: The Recovery Deficit: A Simulation Model of Physician Performance Under Sleep Deprivation

**DOI:** 10.7759/cureus.c445

**Published:** 2026-06-02

**Authors:** Joshua Moen

**Affiliations:** 1 School of Community Medicine, University of Oklahoma Health Sciences Center, Tulsa, USA

This article has been corrected to update the URL for the Medical Performance Sleep Calculator in line 15 of the Results.

